# High-Throughput Drug Stability Assessment via Biomimetic Metalloporphyrin-Catalyzed Reactions Using Laser-Assisted Rapid Evaporative Ionization Mass Spectrometry (LA-REIMS)

**DOI:** 10.3390/pharmaceutics16101266

**Published:** 2024-09-27

**Authors:** András Marton, Zsombor Mohácsi, Balázs Decsi, Balázs Csillag, Júlia Balog, Richard Schäffer, Tamás Karancsi, György Tibor Balogh

**Affiliations:** 1 Department of Chemical and Environmental Process Engineering, Budapest University of Technology and Economics, Műegyetem rakpart 3, H-1111 Budapest, Hungary; amarton@edu.bme.hu (A.M.); balazsdecsi@edu.bme.hu (B.D.);; 2 Waters Research Center, H-1031 Budapest, Hungarytamas.karancsi@ambimass.hu (T.K.); 3 Department of Pharmaceutical Chemistry, Semmelweis University, Hőgyes Endre. u. 9, H-1092 Budapest, Hungary; 4 Center for Pharmacology and Drug Research & Development, Semmelweis University, Üllői út 26, H-1085 Budapest, Hungary; 5 Waters Corporation, Cambridge, MA 02142, USA; julia_balog@waters.com; 6 Ambimass Kft, Záhony u. 7, H-1031 Budapest, Hungary

**Keywords:** high-throughput screening, metabolic stability, ambient mass spectrometry, LA-REIMS, biomimetic metalloporphyrin, lead profiling, in vitro models drug metabolism

## Abstract

**Background:** Building extensive drug candidate libraries as early in the development pipeline as possible, with high-throughput in vitro absorption, distribution, metabolism, and excretion (ADME) profiling, is crucial for the selection of lead compounds to guide subsequent research and production phases. Traditionally, the analysis of metabolic stability assays heavily relies on high-throughput LC-MS/MS (liquid chromatography tandem mass spectrometry) techniques to meet with the lead profiling demands. Laser-assisted rapid evaporative ionization mass spectrometry (LA-REIMS) is a quick and efficient technique for characterizing complex biological samples without laborious sample preparation. **Objective:** In this study, using an automated LA-REIMS well plate reader, achieving an 8 s per sample measurement time, the oxidative metabolic stability of active drug agents was assessed using biomimetic metalloporphyrin-based oxidative model reactions. **Results:** The results obtained using the novel LA-REIMS-based protocol were compared to and corroborated by those obtained using conventional HPLC-UV-MS (high performance liquid chromatography with ultra-violet detection coupled with mass spectrometry) measurements. **Conclusions:** LA-REIMS emerges as a promising technique, demonstrating potential suitability for semi-quantitative high-throughput metabolic stability in an optimized solvent environment.

## 1. Introduction

The rapid identification of optimal pharmacokinetic characteristics plays a pivotal role in drug research. To achieve this, in vitro absorption, distribution, metabolism, and excretion (ADME) screening assays have been extensively developed and employed. These assays generate data that were shown to be predictive of a compound’s pharmacokinetic properties in both preclinical species and humans [1,2]. During the drug discovery process and lead optimization, researchers face the challenge of conducting a multitude of experiments within a limited timeframe, due to the widespread application of different high-throughput screening (HTS) techniques, with the potential to evaluate up to 100,000 individual compounds in just a few days or weeks. The extensive utilization of preclinical animals in vivo to execute these experiments raises not only the specter of exorbitant development costs but also the ethical quandary of aligning with the escalating societal and regulatory expectations regarding the minimization of animal testing in pharmaceutical research [3]. In addition, preparing samples from blood, urine, or tissues is time-consuming, and the metabolites can be at extremely low concentrations, while the biological matrix is a confounding factor that can make analysis very difficult. However, information about the enzymatic transformations that the compounds under study undergo in the body is necessary for both the development and the authorization process. For this reason, there is an increasing interest in in vitro systems that allow for the testing of the solubility and solution stability of drugs in simulated gastrointestinal environments and the absorption of active substances in various epithelial cell membranes [4], as well as being able to replicate the metabolic pathways of active substances without the complexities of a whole physiological context [5].

In 1979, Groves et al. [6] were the first to study synthetic metalloporphyrins in biomimetic oxidation reactions. Using a meso-tetraphenylporphyrin iron(III)–iodosylbenzene system, they modeled hydroxylation and epoxidation reactions of simple alkanes and olefins catalyzed by cytochrome P450 (CYP450) in dichloromethane solution. A myriad of successful applications followed, mimicking various types of oxidation reactions using metalloporphyrins as catalysts [7]. Metalloporphyrins have also been employed in investigations of drug metabolism and the synthesis of metabolites, showcasing their versatility in biochemical research [8,9]. Recently, as model systems for drug metabolism, metalloporphyrins have been utilized in immobilized forms as well, linked to magnetic nanoparticles [10], liver-on-a-chip nanoparticles [11], or in continuous-flow packed-bed reactors [10].

One crucial aspect influencing the activity of metalloporphyrins is their pH sensitivity. Wolak et al. examined the catalytic mechanism of meso-tetrakis(2,4,6-trimethyl-3-sulphonyloxyphenyl) porphyrin-iron(III) dihydrate (FeTPPS(2,4,6-Me)) in relation to the chemical properties of the medium. They found that pH significantly affects the selectivity and the rate of catalyst degradation [12]. A FeTPPS(2,4,6-Me) porphyrin complex as a redox partner promotes the homolytic O-O bond cleavage of a peroxide bond containing oxidants when the pH is <3.5 and >7, which is analogous with the mitochondrial one-electron oxidation within living systems. Between pH 4 and 7, the heterolytic O-O bond cleavage is the thermodynamically favored mechanism. This process corresponds to the two-electron oxidation reaction catalyzed by the microsomal CYP450 enzyme system, which also occurs physiologically [13]. Another key issue in the application of synthetic metalloporphyrin-based systems is the rapid degradation of the catalyst in homogeneous oxidative media. However, the rate of degradation is highly dependent on the structure, the reaction medium, and the quality of the oxidant used [14].

Advancements in label-free, high-throughput mass spectrometry (MS) analysis have significantly contributed to the development of efficient screening of compound libraries. Notably, ambient ionization techniques like acoustic mist ionization (AMI, 2–3 samples/s) [15], desorption electrospray ionization (DESI, 2.7 samples/s) [16], electrospray ionization (ESI, 0.4 samples/s), acoustic droplet ejection (ADE, 0.45 samples/s) [17], and conventional matrix-assisted laser desorption/ionization (MALDI, 2.5 samples/s) [18], as well as hybrid techniques such as infrared matrix-assisted laser desorption electrospray ionization (IR-MALDESI 0.5–1.3 samples/s) [19] and trap-and-elute techniques such as Agilent RapidFire [20], have shown promising results in terms of analytical speed. Despite their clear advantages over traditional label-based photometric methods, fast MS-based applications remain relatively underutilized.

Rapid evaporative ionization mass spectrometry (REIMS) is an ambient ionization technique, initially pioneered for use in the clinical environment for the real-time in situ classification of biological samples and human tissue types [21]. REIMS involves the direct analysis of samples by generating mobile aerosol particles through rapid energy transfer (electric current and laser ablation). The generated sample aerosol is introduced into the REIMS ion source by the suction of the MS vacuum. In the REIMS source, the sample aerosol collides with a heated coil surface, causing the disintegration of the droplets and the generation of ionic species suitable for mass spectrometric detection. As the sampling is achieved through the transfer of mobilized aerosol without application of any valves or frits in the flow path that can be contaminated or blocked easily, its applicability is not dependent on sample consistency. Solid samples and heterogenous or clear solutions can be analyzed using REIMS without significant risk of signal loss due to clogging. REIMS is a soft ionization technique which does not induce in-source fragmentation. Its ability to translate molecular information from mobilized aerosol samples to mass spectral data points offers solutions for a wide range of applications. Coupling REIMS with laser powered sample mobilization (OPO; Optical Parametric Oscillator laser source) and automatic sampling devices transformed the technique into an advanced sampling platform. LA-REIMS (laser-assisted REIMS) has already been used in versatile areas such as salivary metabolomics, [22] microbiology, [23] metabolic biofluid phenotyping, [23] and food authentication [23]. LA-REIMS was also successfully used as a metabolomics platform in cervical cancer screening [24] and for the characterization of canine and feline breast tumors and their metastases [25].

In this study, we report the applicability of automated LA-REIMS in early-stage, high-throughput drug screening, using synthetic metalloporphyrin-based biomimetic oxidation reactions as an in vitro model for CYP450-based metabolism, as an example. Selecting a large and relevant compound set, the performance of the approach was compared to that of traditional HPLC-UV-MS methodology. Although LA-REIMS can also be used for the real-time monitoring of drug consumption and metabolite formation, here we will limit our focus to the assessment of metabolic stability.

## 2. Materials and Methods

### 2.1. Materials

Analytical-grade chemicals were used in the experiments. Methanol, acetonitrile, acetic acid, sodium dihydrogen phosphate, sodium hydrogen phosphate, disodium hydrogen phosphate, *tert*-butyl hydroperoxide (*t*BuOOH), which was used as a co-oxidizing agent for metalloporphyrin, and drug substrates were purchased from Merck Kft (Budapest, Hungary). The catalyst meso-Tetrakis-(sulfonyloxyphenyl)iron porphyrin (FeTPPS) was purchased from Frontier Scientific Inc. (Logan, UT, USA). The formic acid used for analytical measurements was purchased from Alfa Aesar (Ward Hill, MA, USA). The water used for the measurements was produced using a Milli-Q water purification system (Millipore, Bedford, MA, USA).

### 2.2. Sample Preparation

**Figure 1:**
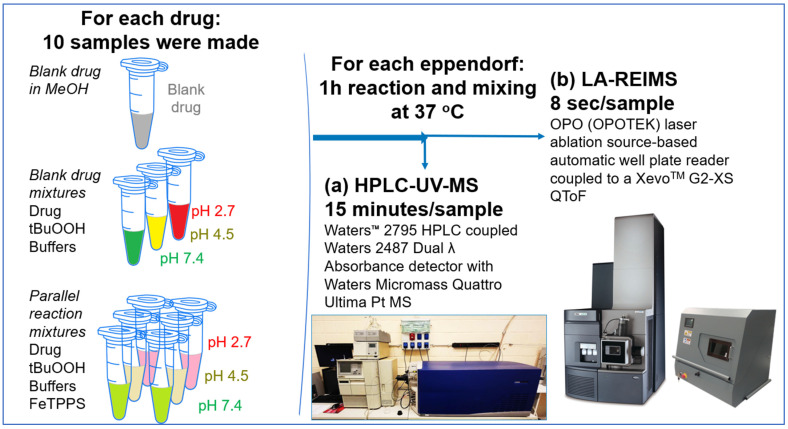
Experimental workflow.

Ten different experiments were assembled for each considered drug molecule (Figure 1): the investigated compound dissolved in methanol (1 mg/mL) serving as a reference for mass accuracy (QC sample), 3 blanks at three different pHs, and 6 reaction mixtures (2 parallels for each pH). Reactions were undertaken at pH 2.7, pH 4.5, and pH 7.4. Blank reaction mixtures were assembled with all components except for the biomimetic catalyst.

The experiments were performed in 1.5 mL microcentrifuge tubes (Eppendorf^®^ Safe-Lock, Hamburg, Germany). The total volume of each reaction mixture (and the blanks as well) was 0.5 mL. When preparing the reaction mixtures, 10 μL of 10 mM drug stock solution and 370 μL of methanol was transferred into each tube. In the case of the biomimetic samples, 13.6 μL of FeTPPS (0.735 mM, dissolved in methanol) solution was added to this mixture, followed by 100 μL of aqueous buffer solution (64 mM) to set the pH (pH 2.7: formic acid, pH 4.5: acetic acid with sodium acetate; pH 7.4: monosodium- and disodium-phosphate) and 6.8 μL of *t*BuOOH stock solution (147 mM). The proportions of the porphyrin/substrate/*t*BuOOH components in the final reaction mixtures were 1/10/100. The final concentration of the drug molecules was 200 µM in each sample tube.

### 2.3. LA-REIMS Well Plate Reader

For this work, we utilized a custom-made LA-REIMS platform prototype which was designed for sampling from well plates. This LA-REIMS prototype was used for research purposes only. The LA-REIMS technique was based on a direct analysis of the aerosol generated by laser ablation (OPOTEK OPO laser, λ = 2940 nm, 5 mJ/pulse, 20 Hz shot frequency, 6 ns pulse width). The generated aerosol droplets were introduced into the REIMS atmospheric interface, where the ion generation occurred via collision with a high-temperature impactor surface. For focusing the ablation beam, an uncoated spheric calcium fluoride lens with a 50 mm focal length was used. Motorized 3D (three dimensional) stages (Thorlabs, Newton, NJ, USA) were used to position the well plates in X–Y–Z directions. Raw data were acquired using a Xevo^TM^ G2-XS TOF-MS (Waters Corporation, Milford, MA, USA); the spectra were acquired in positive ion mode over a mass-to-charge range of 50–1200 with 0.5 s/scans. Leucine enkephalin was used as a lock mass material. Data were processed using Waters MassLynx^TM^ 4.2 software.

### 2.4. HPLC-UV-MS Measurements

The HPLC-UV-MS experiments were performed using a Waters Micromass^TM^ Quattro Ultima Pt tandem quadrupole mass spectrometer coupled to a Waters 2795 liquid chromatography system (Milford, MA, USA). Detection was performed with a Waters 2487 Dual λ Absorbance detector. The analysis was performed at 40 °C on a Kinetex XB C18 column (150 × 4.6 mm, 2.6 µm) (Phenomenex, Torrance, CA, USA) at a mobile phase flow rate of 1 mL/min. The eluent A composition used was 0.1 vol % formic acid dissolved in water and eluent B was a 95:5 mixture of MeCN:H_2_O to which 0.1 vol % formic acid was added. The gradient elution program proceeded as follows: a linear gradient of 5–100% B was applied in the range 0–11 min, and an isocratic phase of 100% B was applied in the range 11–13 min. This was followed by a 2 min equilibration period before the next injection using a 5% B composition. The injection volume was adjusted to 5 µL and the chromatographic profile was recorded at a wavelength of λ = 220 (±4) nm. The mass spectrometry conditions were as follows: ion source ESI (electrospray ionization), positive ion mode, scanning ion mode (150–600 *m*/*z*), drying gas (N2) temperature of 350 °C, flow rate of 5.5 L/min, nebulizer gas (N2) pressure of 6 bar, quadrupole temperature of 120 °C, capillary voltage of 2500 V, and fragmentor voltage of 60 V. Data were processed using MassLynx^TM^ 4.0 software.

## 3. Results and Discussion

### 3.1. Experimental Methodology

First, a diverse compound library of commercially available active drug agents was selected, which are relevant and representative of different drug classes (76 drugs, listed on the Supplementary Materials). Then, the metabolic stability assays were prepared using synthetic metalloporphyrin-based biomimetic oxidation reactions to imitate CYP450-based metabolism. The reactions were performed at various pH levels to study the Ph-dependent oxidative behavior of synthetic metalloporphyrin. For the high-throughput measurements, an automated LA-REIMS well plate reader was utilized (8 s per sample measurement time). The results were obtained using conventional HPLC-UV-MS (15 min per sample measurement time), as well using a standard protocol for metabolic stability assessment. The conversion rates were calculated by comparing the peak area of the appropriate compound in the selected ion chromatogram before and after the reaction. Finally, appropriate statistical methods were interpreted to assess the regression, correlation, and agreement between the results of analytical techniques to confirm that the LA-REIMS is a feasible alternative for high-throughput ADME profiling in drug discovery.

### 3.2. Biomimetic Sample Evaluation and Data Acquisition

We studied the biomimetic stability of a 76-member, diverse compound library of commercially available active drug agents at three different pHs: pH = 2.7, pH = 4.5, and pH = 7.4. The reaction solutions contained the drug substance, the oxidizing agent (tert-butyl hydroperoxide), the metalloporphyrin catalyst, and the necessary buffers.

**Figure 2:**
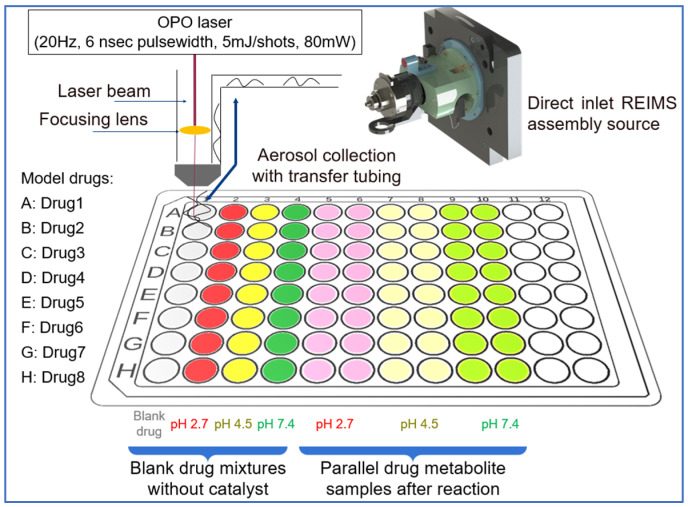
Scheme of the LA-REIMS measurement setup.

The prepared solutions were pipetted onto the well plate. All samples for each drug molecule were arranged in a separate row on the well plate (Figure 2). In this experimental arrangement, each well was filled with 100 µL of reaction blank or reaction mixture solutions. LA-REIMS data acquisition (with an 8 s/sample measuring time) was conducted row-by-row, with one raw file containing all ten data points for a given active pharmaceutical ingredient.

**Figure 3:**
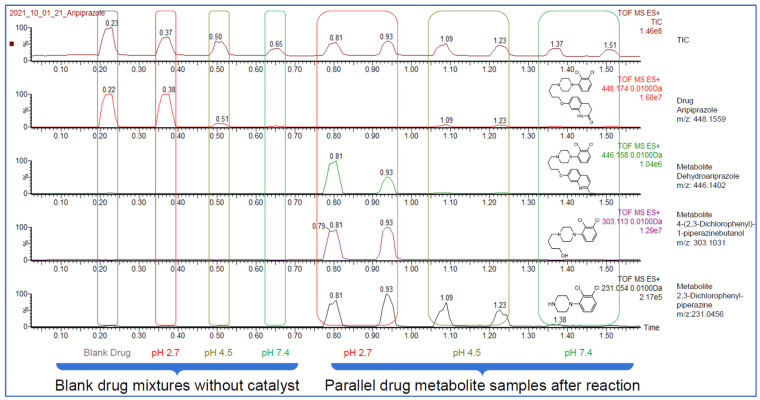
Example of the LA-REIMS-acquired data. Chromatograms of the 10 samples of Aripiprazole model drug were acquired within 1.5 min. The first trace in the figure shows the total ion chromatogram (TIC), while the second trace displays the ion chromatogram specific to aripiprazole. The bottom three traces illustrate examples of proposed metabolites. Users can extract and display the required peaks as an extracted chromatogram. The pH dependency of the metabolite formulation and oxidative metabolism is visible from the chromatograms (TIC: total ion chromatogram).

Figure 3 illustrates the results obtained through the analysis of aripiprazole, as a representative compound. The acquisition of the ten independent data points required only 1.5 min. It is clearly visible from the extracted ion chromatogram (second trace from the top) that the degradation of aripiprazole was nearly complete at pH = 2.7, while at pH = 4.5 and pH = 7.4, the degradation was not significant. The appearance of the major degradation products can also be observed, as it is illustrated on the bottom three traces of Figure 3, without requiring any additional post-analysis measurements. Obtaining similar results for this compound using a conventional HPLC-UV-MS would have taken a few hours. Our protocol remained stable throughout the measurement. The screening of the set of 76 drug molecules required a total of 1 h 42 min runtime.

The ion intensity of the aripiprazole decreased as the pH increased, as shown in Figure 3 (second trace from the top, with the blank mixtures 2, 3, and 4 peak positions from the left). This decrease can be attributed to the pH-dependent ionization efficiency and/or the matrix effect caused by the different buffer systems used in the LA-REIMS technique. Notably, in this technique, the compound of interest was not separated from the matrix, which could influence the observed ion intensity and therefore the detection sensitivity of certain metabolites. This is an important consideration when evaluating the utility of LA-REIMS for drug metabolite characterization. To address this concern, we checked aripiprazole’s metabolite profile in the LC-MS results and these results showed good overall agreement with the metabolite profile acquired by LA-REIMS.

More details for aripiprazole and further examples can be found in the Supplementary Materials.

### 3.3. Regression Comparison of Results Obtained Using LA-REIMS and LC-UV-MS

Conversion rates can be obtained by simply comparing the peak integral of M+ of each molecule in the blank and post-reaction mixtures at their corresponding pHs. Since LA-REIMS is a soft ionization technique, it does not induce in-source fragmentation. Conversion rates measured by LA-REIMS were compared with those obtained using LC-UV and LC-MS setups (only metabolic stability results at pH 4.5 are presented, as this pH level aligns with the CYP450 two-electron oxidation mechanisms relevant to this study. Although MS signals might have exhibited different linearity compared to UV absorbance, we assessed the correlation between the decrease in MS signal intensity and UV absorbance. At pH 7.4, the sensitivity of LA-REIMS was significantly reduced due to ion suppression caused by buffer matrix effects. This sensitivity reduction was acceptable, as, beyond pH 7, the formation of an artificial oxo-dimer deactivated the metalloporphyrin catalyst.)

**Figure 4:**
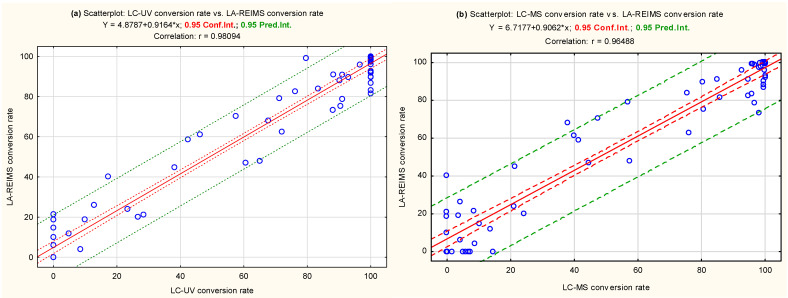
Regression scatter plots comparing the conversion rates measured using LA-REIMS with (**a**) LC-UV and (**b**) LC-MS at pH = 4.5 in positive ion mode. These plots highlight the agreement and correlation between the different analytical methods.

Figure 4 demonstrates strong agreement in drug conversion rates between LC-UV and LA-REIMS, as well as between LC-MS and LA-REIMS at pH = 4.5. The correlation coefficient between LA-REIMS and LC-UV or LC-MS was found to be r = 0.98094 and r = 0.96488, respectively, indicating a strong positive linear relationship. Additionally, the slopes of the regression lines were also comparable, indicating consistent conversion rate relationships across the different analytical methods.

**Figure 5:**
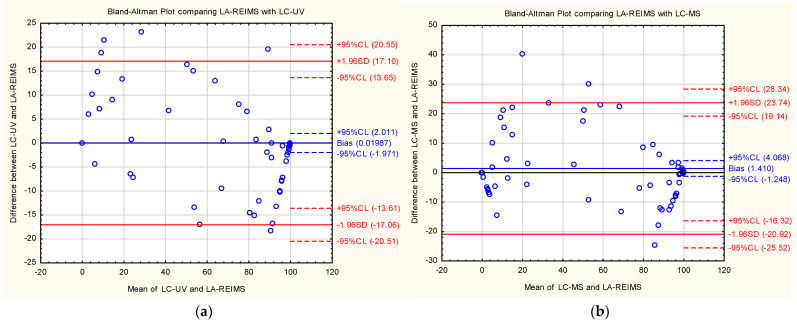
Bland-Altman plots for the conversion rates measured using LA-REIMS, compared with (**a**) LC-UV and (**b**) LC-MS results at pH = 4.5 in positive ion mode.

Bland–Altman plots (Figure 5) are commonly used to assess the agreement between two different instruments or two measurements techniques. In these plots, the X axis represents the means of the compared measurement conversion rates, and the Y axis shows the difference between the two measurements. The plots reveal that our data points were roughly horizontally distributed around the zero line. The scatter appears homogeneous. This also confirms that there was good agreement between the two measurement methods. A small amount of bias appears for both comparisons (0.019 and 1.410). Regarding the trends, LA-REIMS appeared to be a comparable method for measuring the conversion rates, as the variance seemed similar at both endpoints. The limit of acceptance was approximately 17 when comparing with LC-UV and 20.92–23.74 when comparing with LC-MS.

In general, the results obtained with the LA-REIMS workstation showed a strong correlation with those obtained with a conventional HPLC-UV-MS method, while providing a significant reduction in analytical runtime.

### 3.4. Solvent Requirements and Measurement Time

High-throughput screening via LA-REIMS technology can be a greener alternative to traditional LC-based techniques, due to its lower solvent requirement. This reduction in solvent usage not only minimizes its environmental impact but also contributes to cost-effectiveness and sustainability. By requiring a lower volume of solvents, LA-REIMS facilitates an eco-friendlier approach in analytical chemistry, aligning with the growing emphasis on green chemistry practices. Consequently, its integration into high-throughput screening processes not only enhances efficiency but also underscores its role in advancing environmentally conscious analytical methodologies (Table 1).

## 4. Conclusions

In conclusion, LA-REIMS emerges as a promising technique, demonstrating potential suitability for semi-quantitative high-throughput metabolic stability in an optimized solvent environment, which also allows for the characterization of metabolites. Notably, LA-REIMS possesses a distinct speed advantage over conventional HPLC-UV-MS measurements, without compromising sensitivity or incurring significant information loss, while the application of the technique also reduces solvent requirements by several orders of magnitude. LA-REIMS is thus a more environmentally friendly measurement technique that is ideally suited to the modern pharmaceutical industry. LA-REIMS can also facilitate the real-time recording of pharmacokinetic curves directly from a well plate, eliminating the need for sample preparation, which can also support the traditional ADME-based microsomal metabolic stability research. Our future endeavors aim to comprehensively explore microsomal systems leveraging this methodology.

## Figures and Tables

**Table 1:** Comparison of solvent requirements and measurement times of measurement techniques (* UHPLC (Ultra High Performance Liquid Chromatography) results are from the literature and are presented for comparability [26]).

Technique	One Sample	10 Samples (to Characterize a Drug)	760 Samples (Whole Dataset)
HPLC-based	15 mL 15 min	150 mL 150 min	11,550 mL 212.5 h
UHPLC-based *	0.3 mL 1 min	3 mL 10 min	231 mL ~13 h
HTS LA-REIMS	0.02 mL 8 s	0.2 mL 80 s	15.4 mL <1 h 42 min

## Data Availability

Data is contained within the article or Supplementary Materials.

## Supplementary Materials

The following supporting information can be downloaded at: https://www.mdpi.com/article/10.3390/pharmaceutics16101266/s1. Figure S1: Aripiprazole’s reaction blanks and reaction mixture’s metabolic profile at pH 2.7 (extracted ion chromatograms); Figure S2: Aripiprazole’s reaction blank’s and reaction mixture’s metabolic profile at pH 4.5 (extracted ion chromatograms); Figure S3: Aripiprazole’s reaction blank’s and reaction mixture’s metabolic profile at pH 7.4 (extracted ion chromatograms); Figure S4: Domperidone’s reaction blank’s and reaction mixture’s metabolic profile at pH 2.7 (extracted ion chromatograms); Figure S5: Domperidone’s reaction blank’s and reaction mixture’s metabolic profile at pH 4.5 (extracted ion chromatograms); Figure S6: Domperidone’s reaction blank’s and reaction mixture’s metabolic profile at pH 7.4 (extracted ion chromatograms; Figure S7: Extracted LA-REIMS chromatograms of Domperidone; Figure S8: Verapamil’s reaction blank’s and reaction mixture’s metabolic profile at pH 2.7 (extracted ion chromatograms); Figure S9: Verapamil’s reaction blank’s and reaction mixture’s metabolic profile at pH 4.5 (extracted ion chromatograms); Figure S10: Verapamil’s reaction blank’s and reaction mixture’s metabolic profile at pH 7.4 (extracted ion chromatograms; Figure S11: Extracted LA-REIMS chromatograms of Verapamil; Table S1: Used APIs and measured conversion rates on pH 4.5.
