# Supplementary material for: High-Throughput Drug Stability Assessment via Biomimetic Metalloporphyrin-Catalyzed Reactions Using Laser-Assisted Rapid Evaporative Ionization Mass Spectrometry (LA-REIMS)
Source: Pharmaceutics. 2024 Sep 27;16(10):1266. doi: 10.3390/pharmaceutics16101266 (PMC11510429; doi:10.3390/pharmaceutics16101266)
Supplement: Supplementary file 1 [file pharmaceutics-16-01266-s001.zip › pharmaceutics-3208671-supplementary.pdf]

Contents

Model APIs 2
Aripiprazole LC-MS results 2
Domperidon LC-MS, LA-REIMS results 5
Verapamil – LC-MS and LA-REIMS results 9
Used APIs and measured conversion rates on pH 4.5 13

Model APIs

Aripiprazole LC-MS results

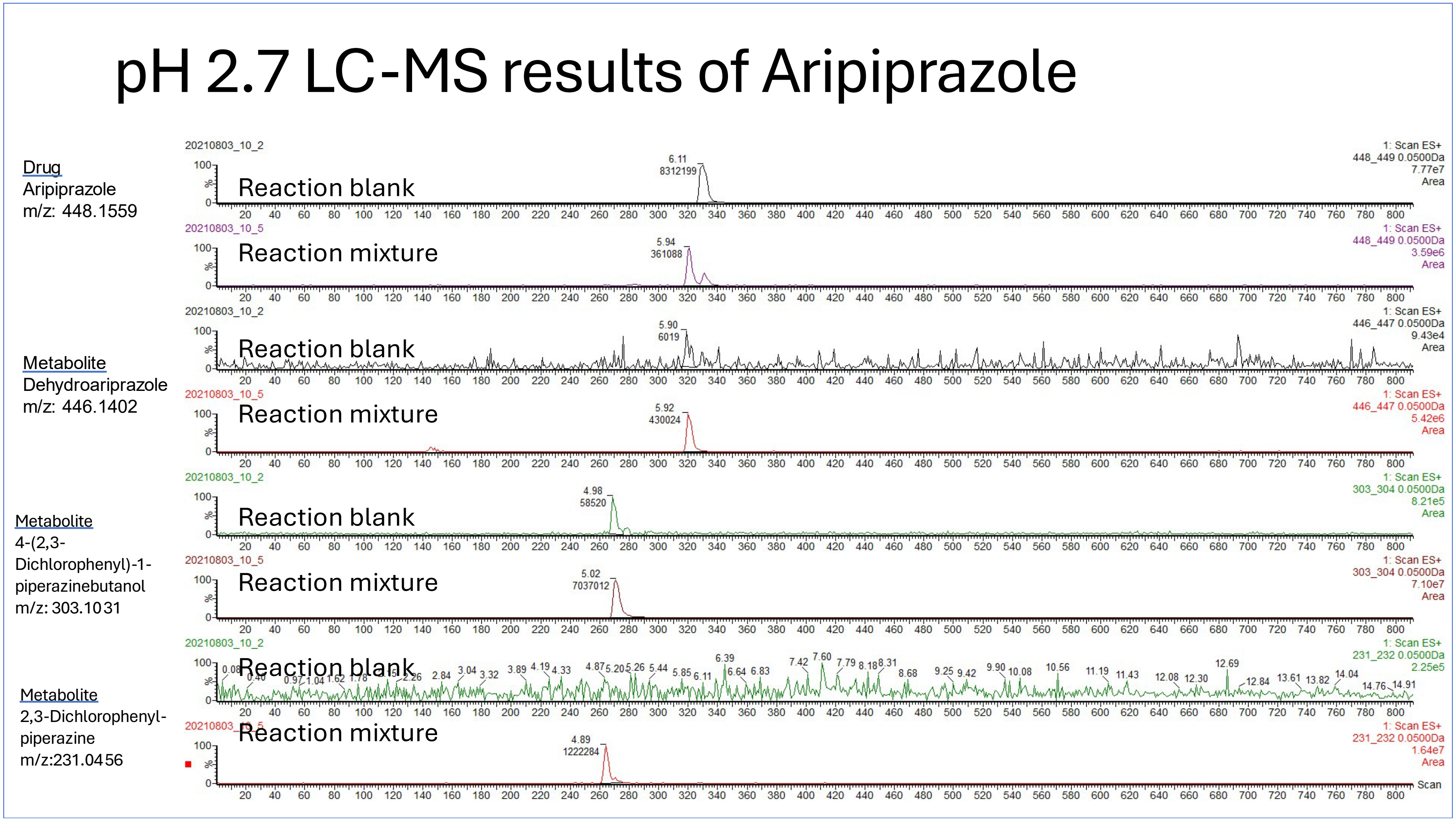

Supplementary Figure S1: Aripiprazole's reaction blanks and reaction mixture's metabolic profile at pH 2.7 (extracted ion chromatograms)

pH 4.5 LC-MS results of Aripiprazole

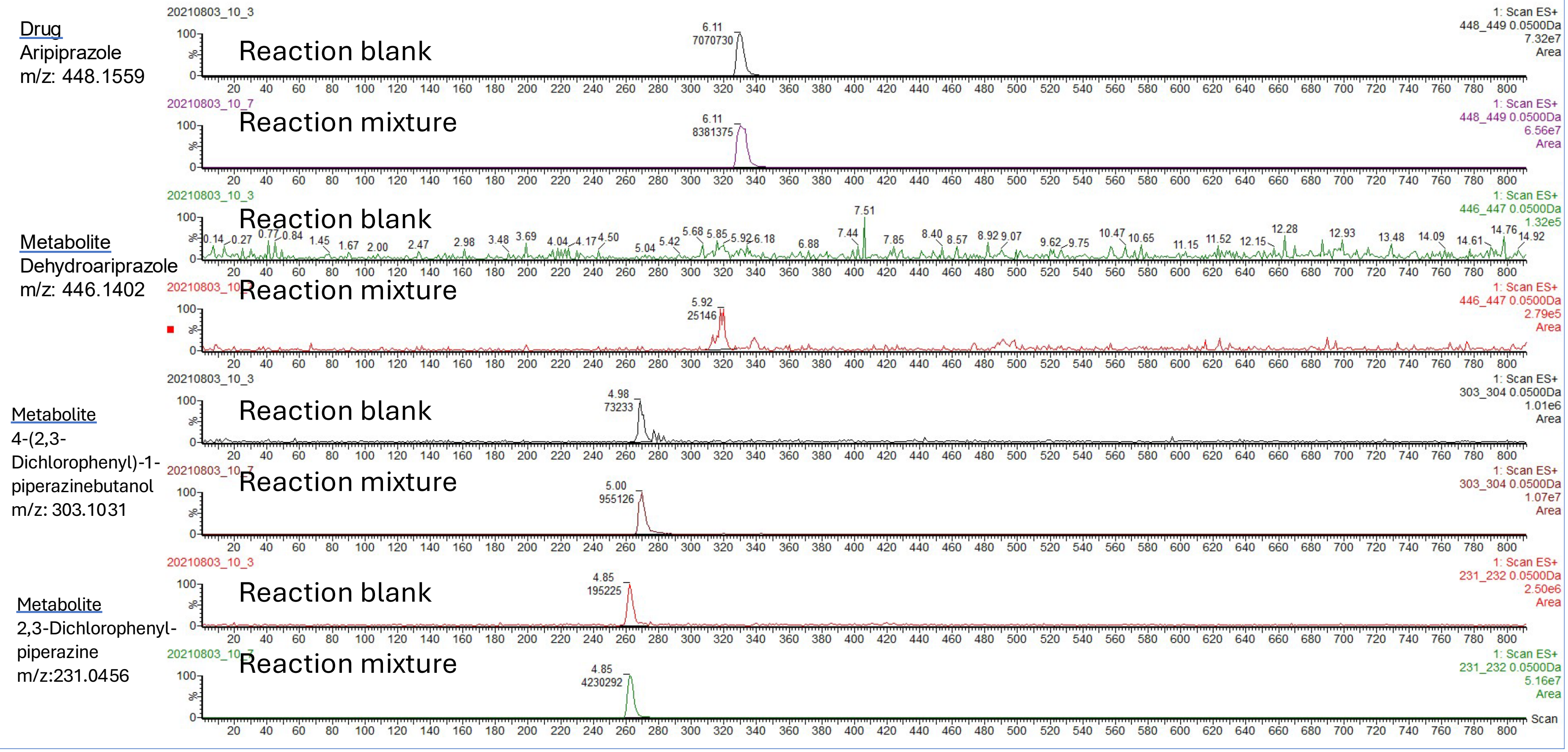

Supplementary Figure S2: Aripiprazole's reaction blank's and reaction mixture's metabolic profile at pH 4.5 (extracted ion chromatograms)

pH 7.4 LC-MS results of Aripiprazole

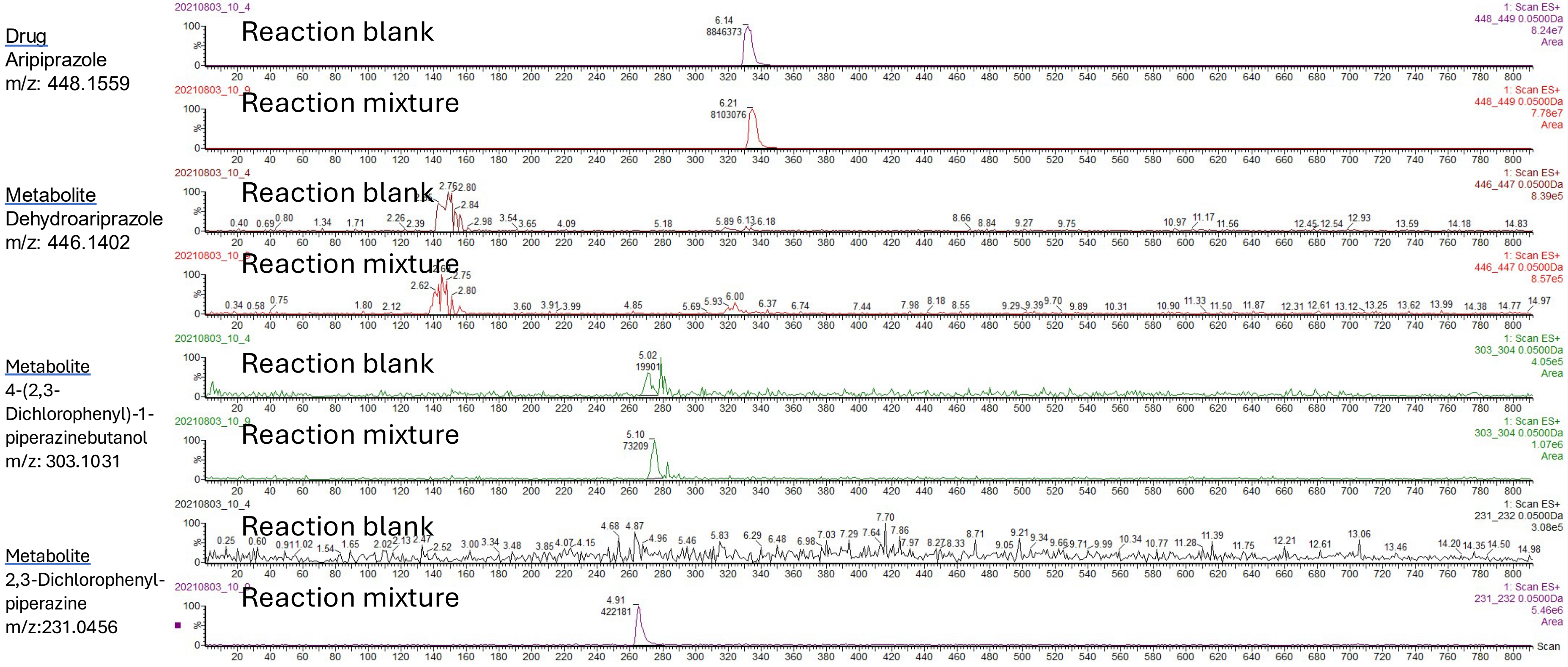

Supplementary Figure S3: Aripiprazole's reaction blank's and reaction mixture's metabolic profile at pH 7.4 (extracted ion chromatograms)

Domperidon LC-MS, LA-REIMS results

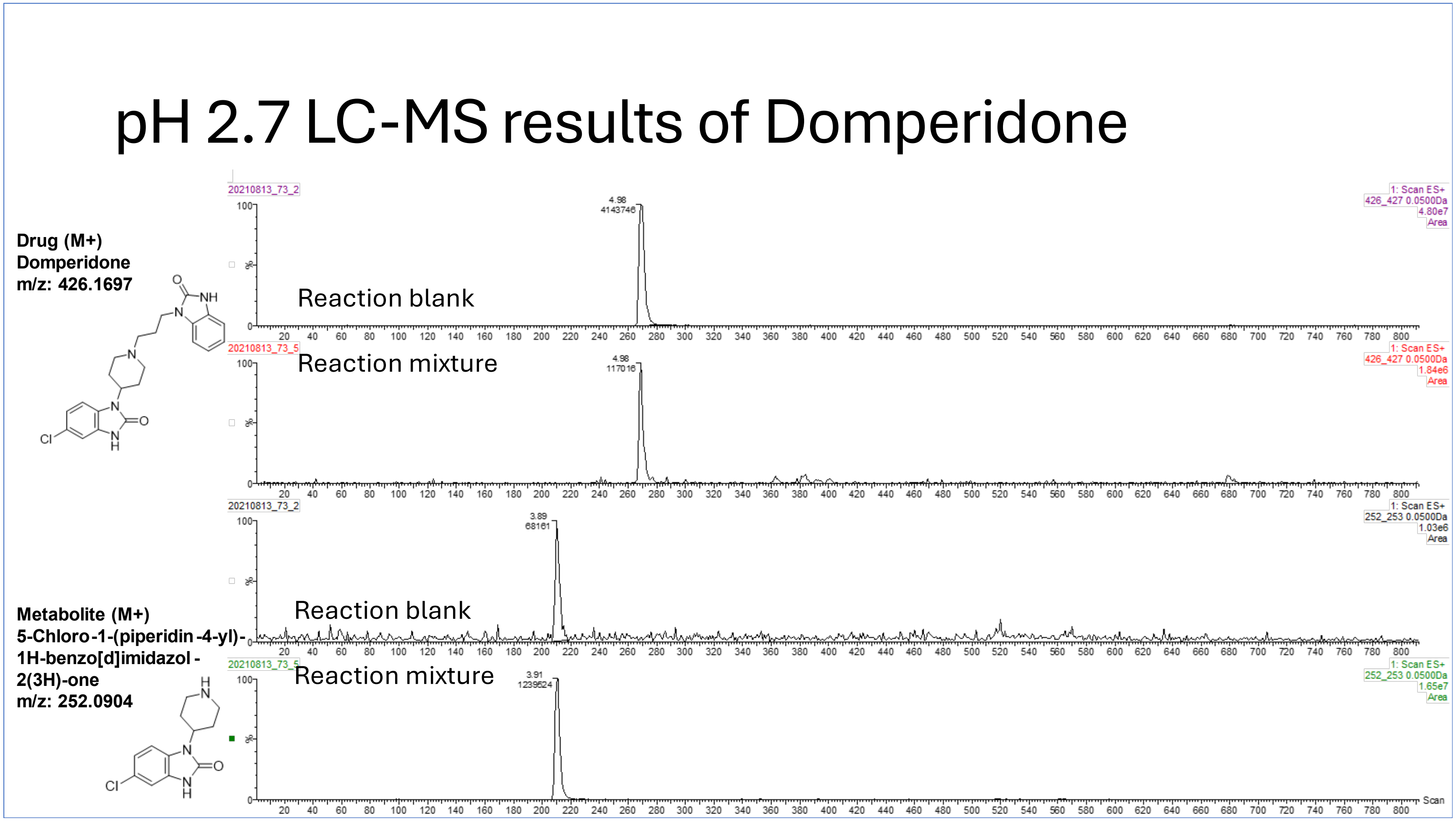

Supplementary Figure S4: Domperidone's reaction blank's and reaction mixture's metabolic profile at pH 2.7 (extracted ion chromatograms)

pH 4.5 LC-MS results of Domperidone

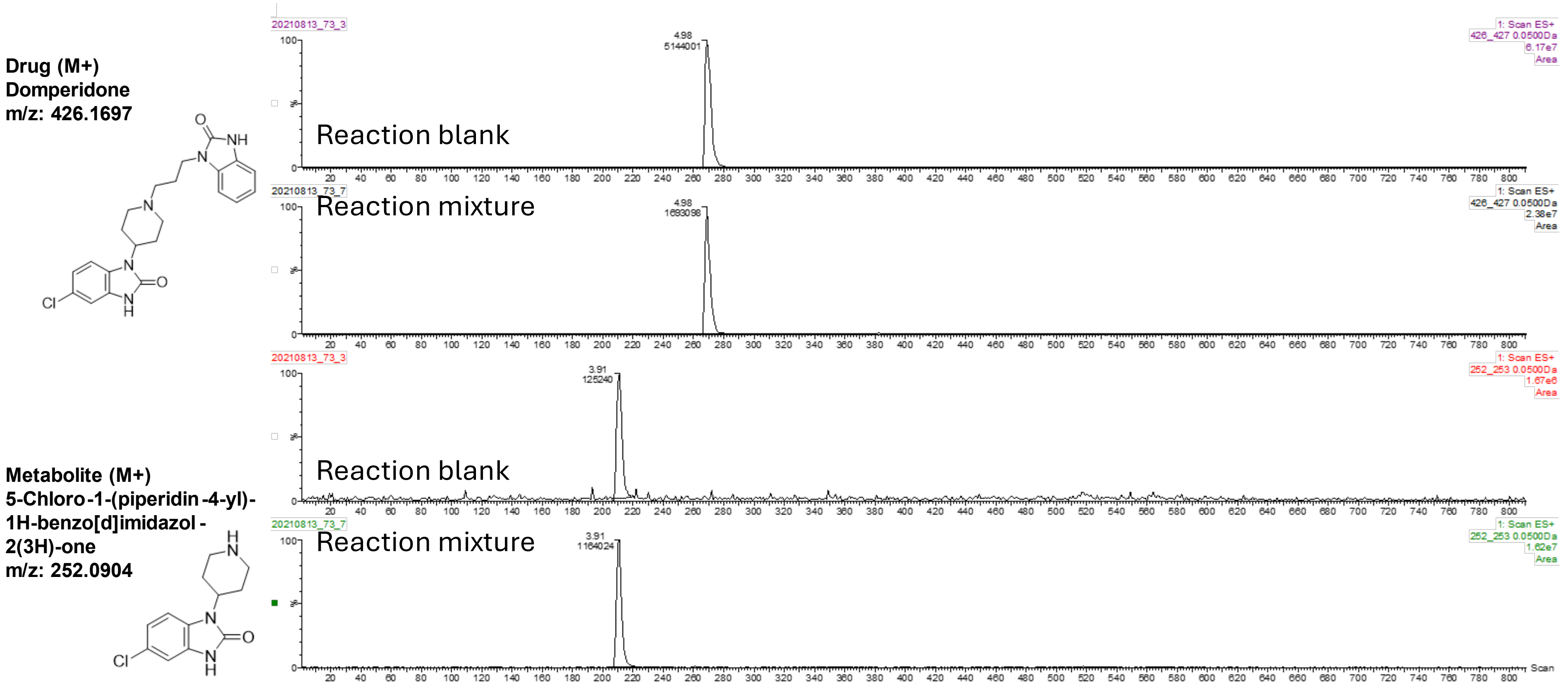

Supplementary Figure S5: Domperidone's reaction blank's and reaction mixture's metabolic profile at pH 4.5 (extracted ion chromatograms

pH 7.4 LC-MS results of Domperidone

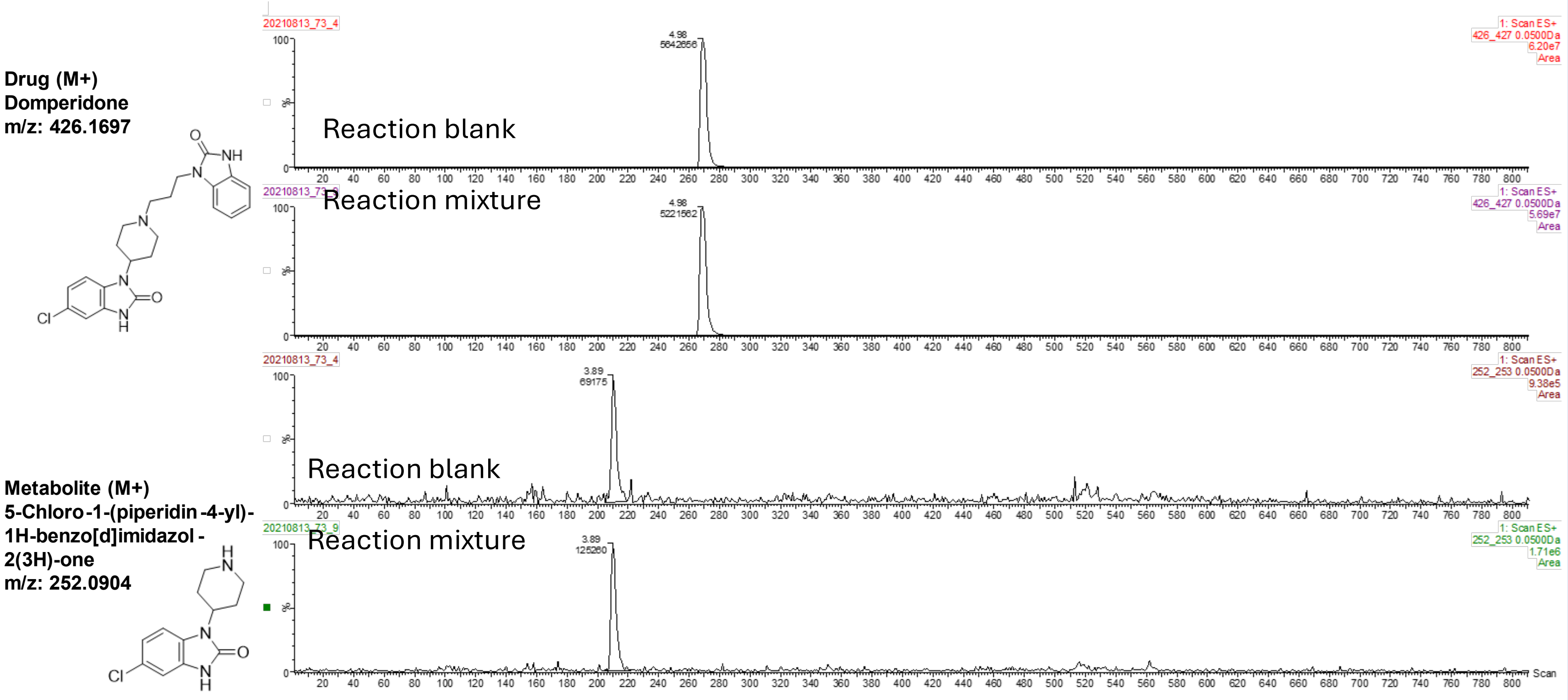

Supplementary Figure S6: Domperidone's reaction blank's and reaction mixture's metabolic profile at pH 7.4 (extracted ion chromatograms

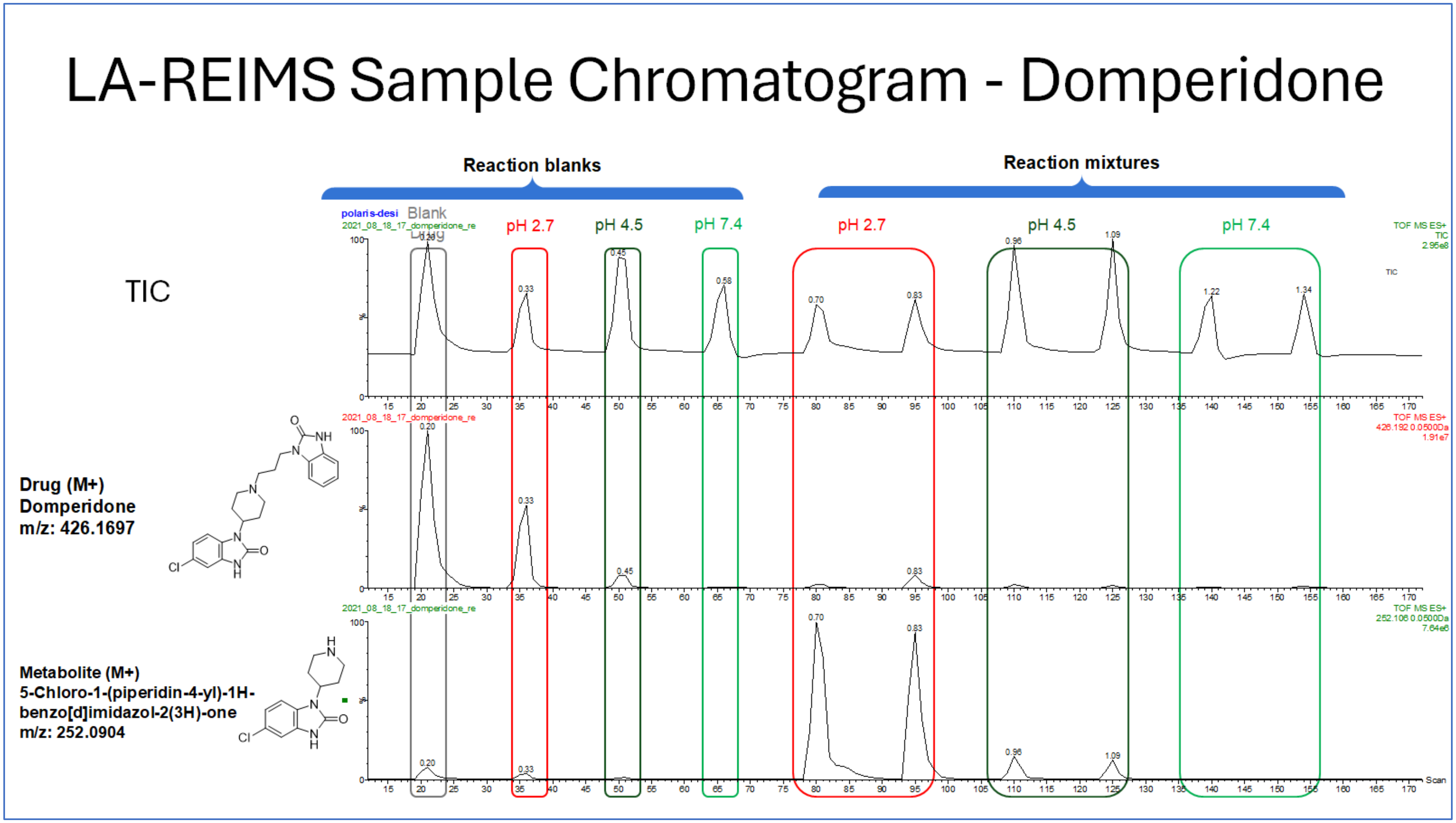

Supplementary Figure S7: Extracted LA-REIMS chromatograms of Domperidone

Verapamil – LC-MS and LA-REIMS results

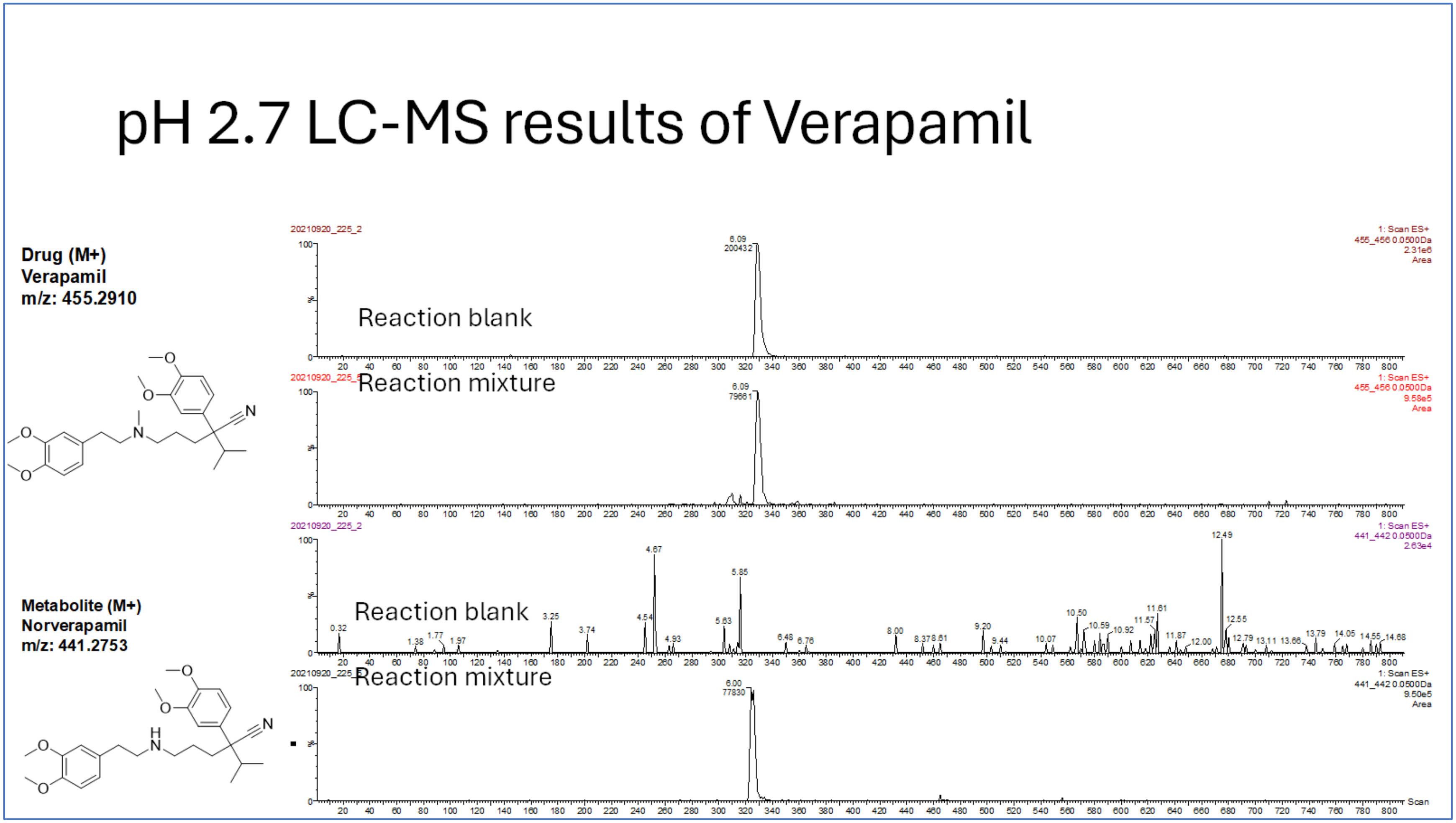

Supplementary Figure S8: Verapamil's reaction blank's and reaction mixture's metabolic profile at pH 2.7 (extracted ion chromatograms

pH 4.5 LC-MS results of Verapamil

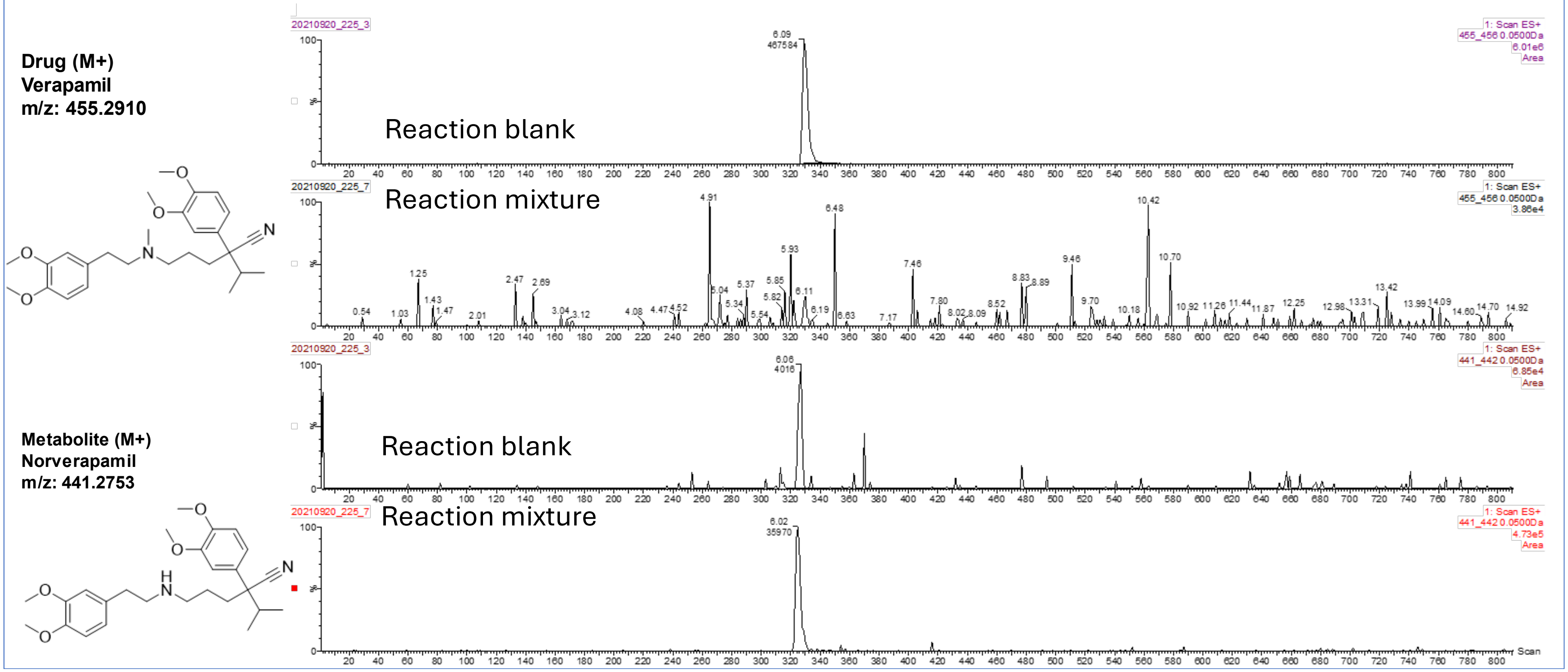

Supplementary Figure S9: Verapamil's reaction blank's and reaction mixture's metabolic profile at pH 4.5 (extracted ion chromatograms

pH 7.4 LC-MS results of Verapamil

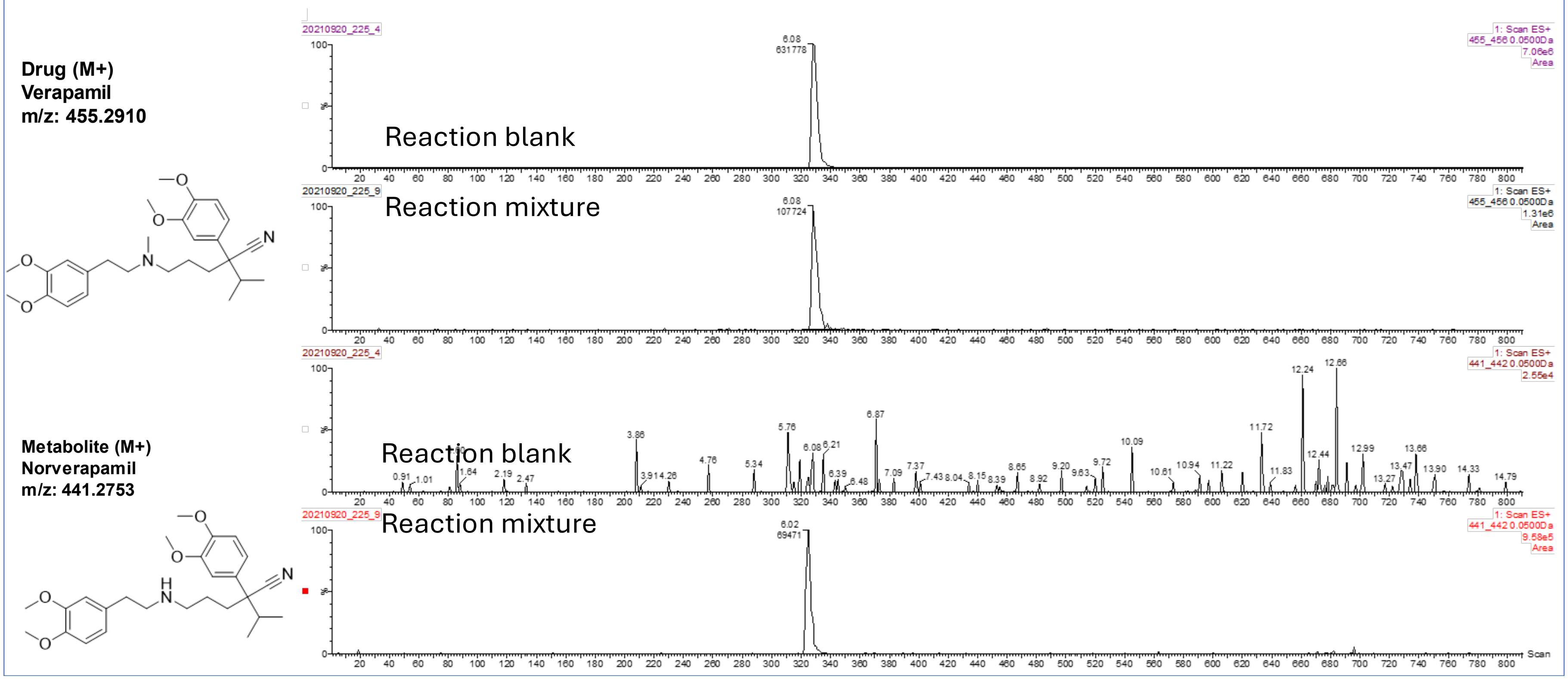

Supplementary Figure S10: Verapamil's reaction blank's and reaction mixture's metabolic profile at pH 7.4 (extracted ion chromatograms

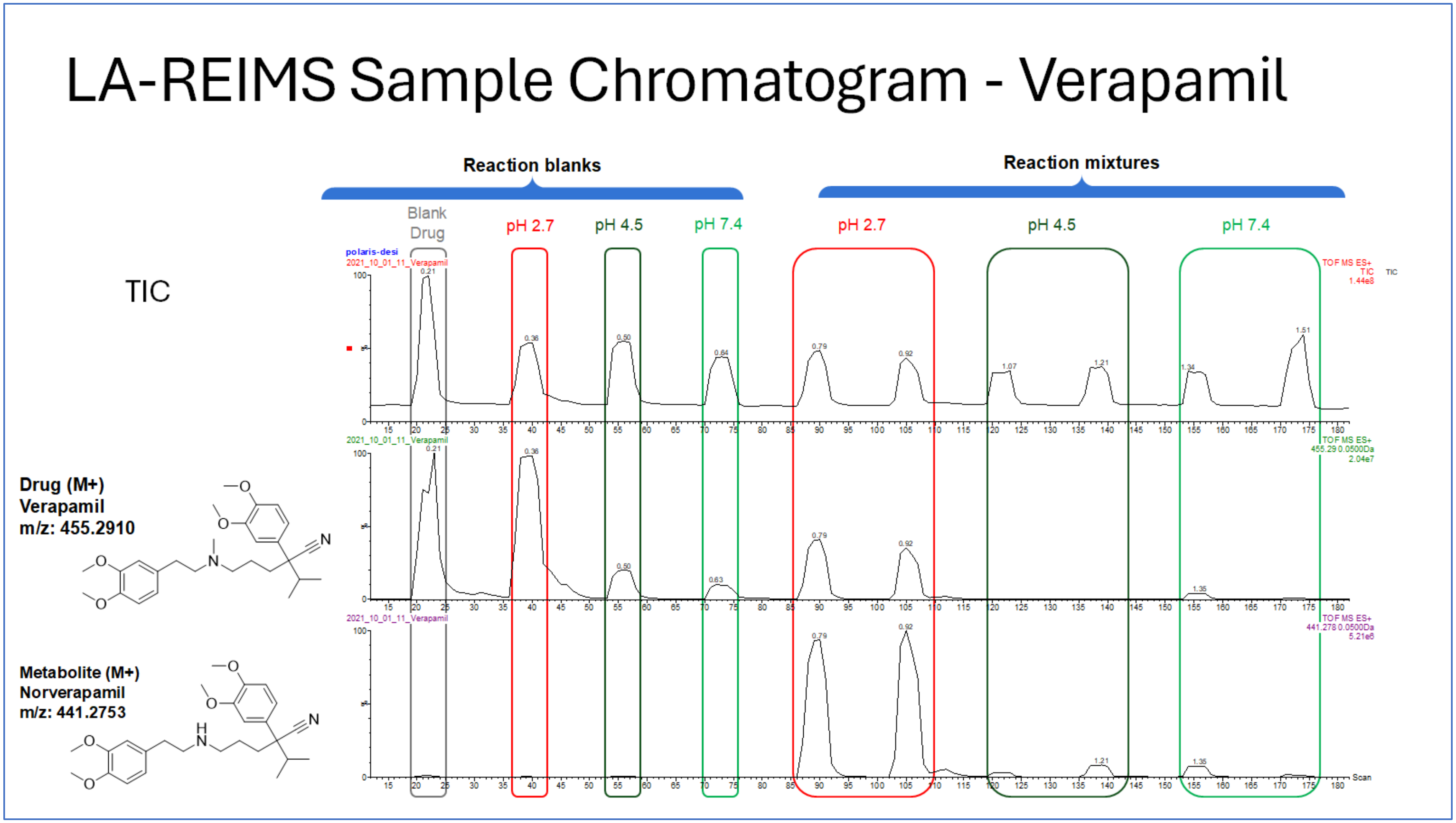

Supplementary Figure S11: Extracted LA-REIMS chromatograms of Verapamil

Used APIs and measured conversion rates on pH 4.5

API name	Structures	Measured conversion rates on pH4.5(%)		
		LC-UV	LC-MS	LA-REIMS
Amodiaquine	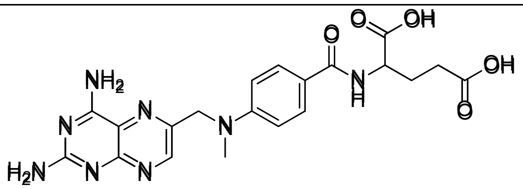	100.00	100.00	99.41
Amlodipine	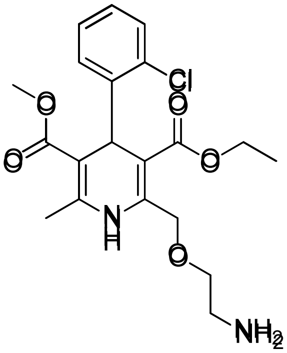	100.00	99.92	100.00
Aripiprazole	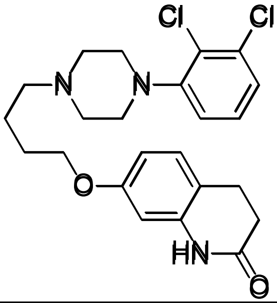	17.20	0.00	40.25
Astemizole	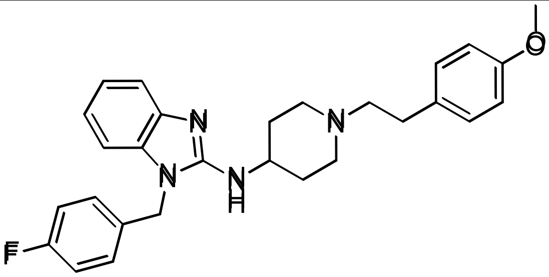	100.00	100.00	99.88
Atorvastatin	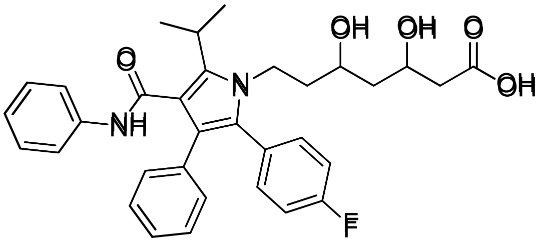	100.00	95.82	83.23

Benidipin	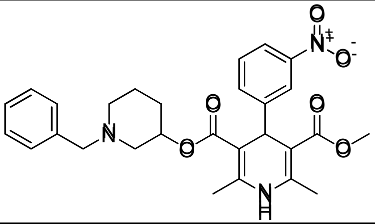	100.00	98.62	98.04
Bepridil	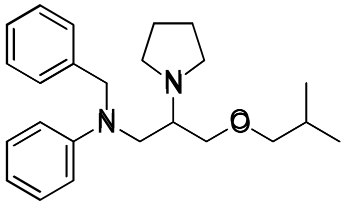	100.00	95.82	99.13
Bifonazole	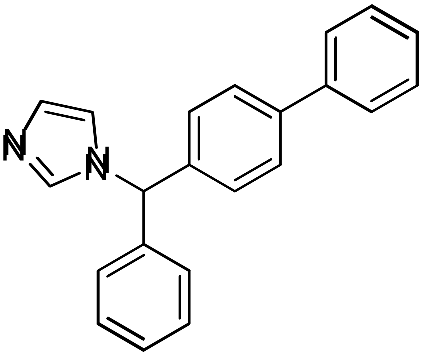	0.00	0.00	0.00
Bromfenac	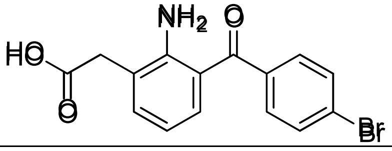	100.00	100.00	99.87
Buspirone	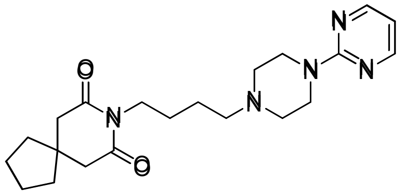	67.69	38.12	68.04
Chloroquine	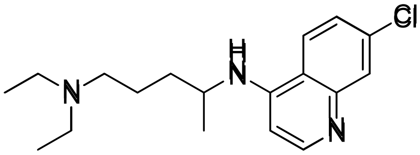	96.55	92.60	95.91
Chlorpromazine	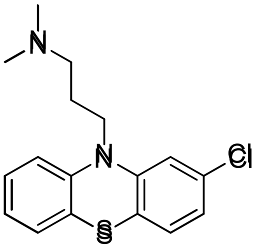	71.18	57.02	79.22

Chlofazimine	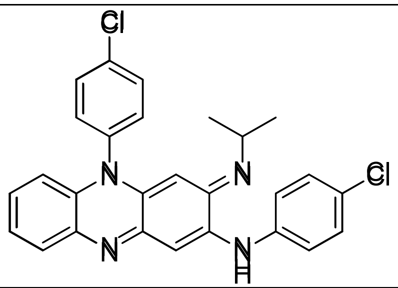	88.04	98.07	73.41
Clopidogrel	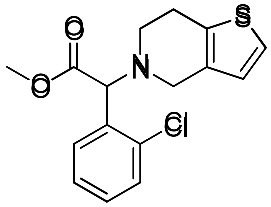	23.34	21.15	24.04
Clotrimazole	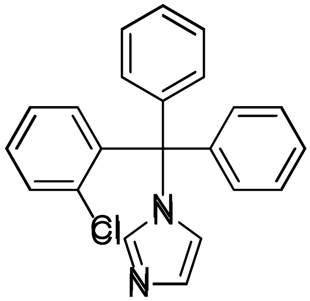	0.00	6.87	0.00
Clozapine	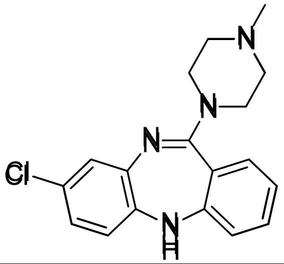	92.88	80.45	89.72
Colhichine	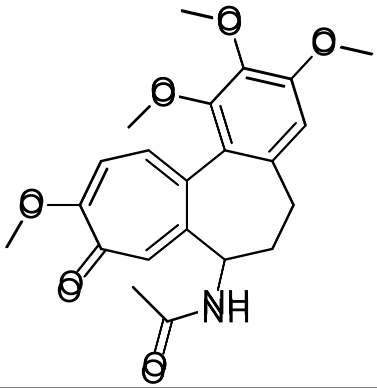	0.00	10.13	14.73
Enalapril	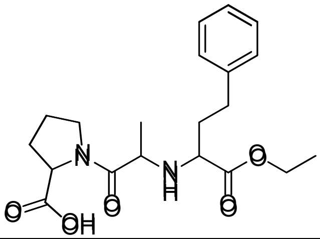	0.00	4.17	6.00

Disopyramide	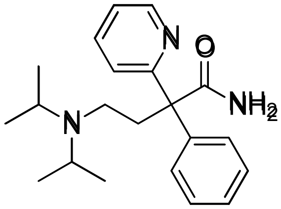	4.81	13.75	11.82
Diloxandine	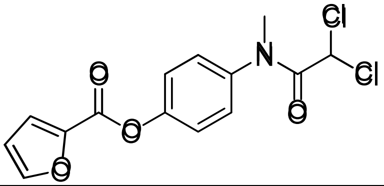	0.00	0.00	0.00
Dilthiazem	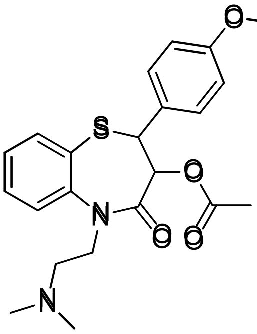	57.49	47.30	70.35
Domperidone	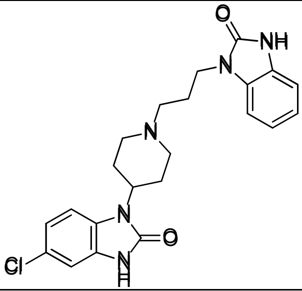	90.46	80.64	75.33
Emetan / emetine	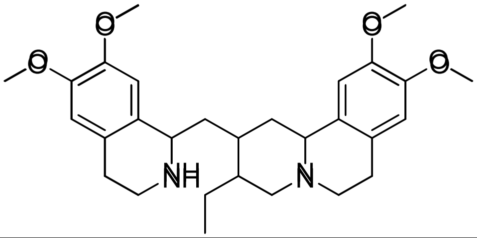	76.17	94.64	82.65
Enoxacin	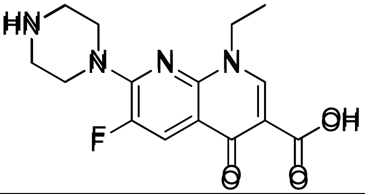	42.39	41.36	58.72
Ethopropazine	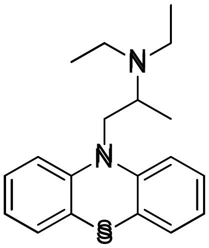	100.00	100.00	92.84

Famotidine	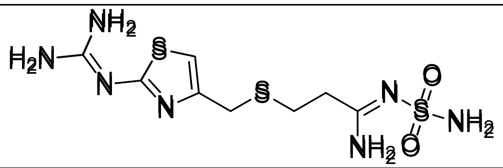	100.00	100.00	91.92
Fenoterol	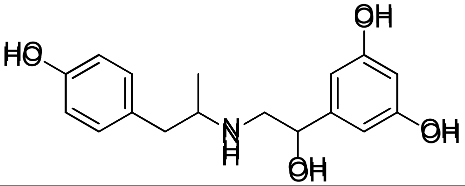	100.00	100.00	100.00
Fentanyl	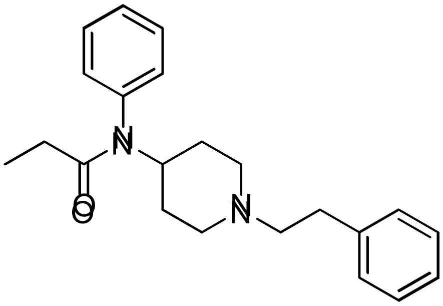	0.00	8.53	21.43
Fexofenadine	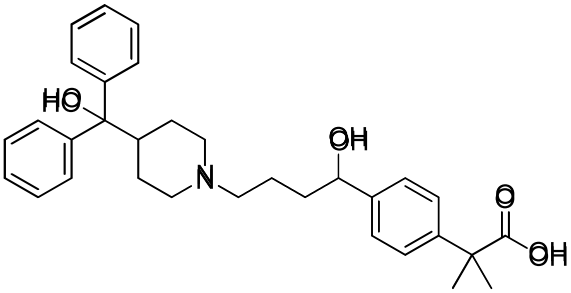	9.86	3.50	18.83
Fluoxetine	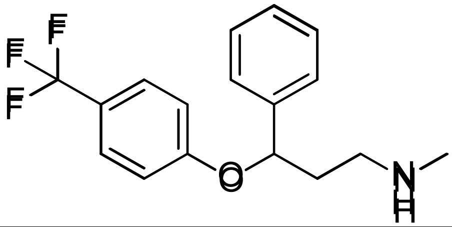	0.00	0.00	0.00
Fluphenazine	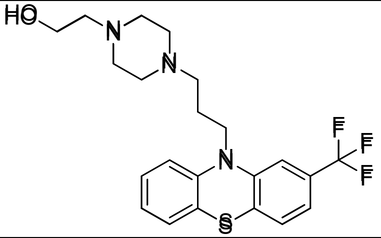	100.00	99.65	89.93
Glybenclamide	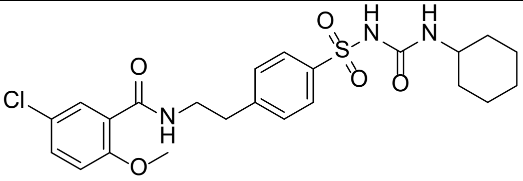	46.14	40.03	61.14

Griseofulvine	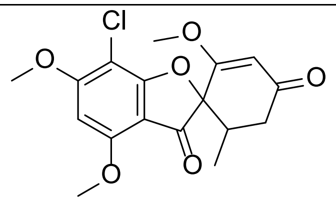	0.00	0.00	0.00
Hydrocortisone	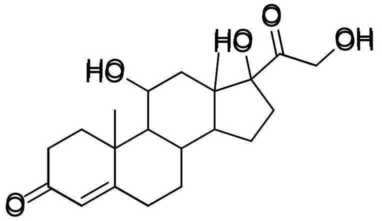	26.59	24.31	20.17
Hydroxyzine	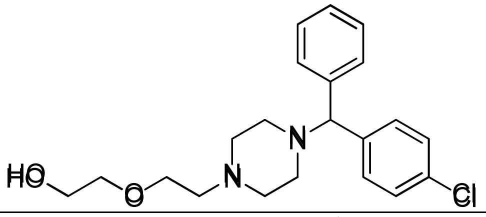	8.48	8.77	4.00
Idarubicin	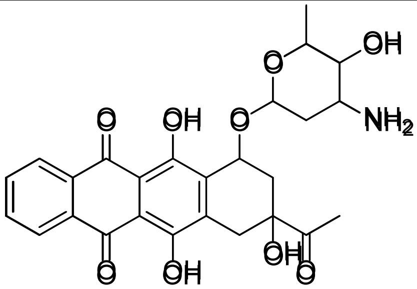	100.00	99.33	99.99
Indoramine	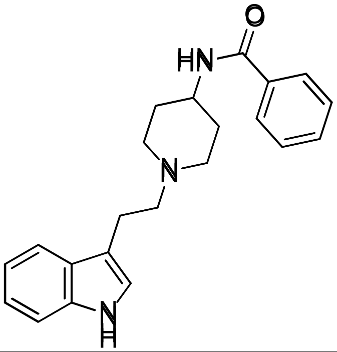	100.00	99.96	100.00
Irbesartan	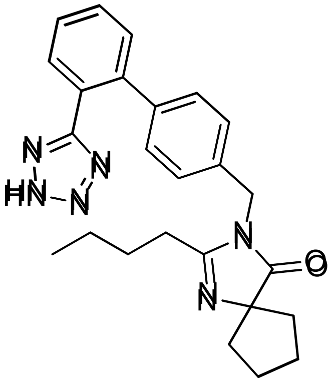	0.00	7.38	0.00

Isoxicam	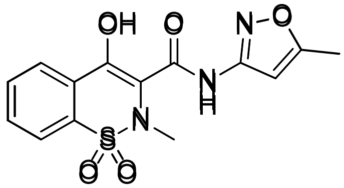	100.00	98.55	99.90
Ketoconazole	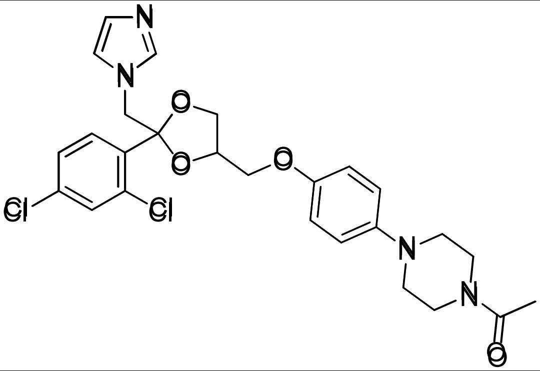	100.00	96.78	98.58
Labetalol	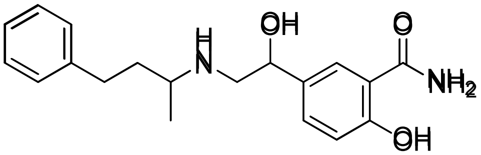	83.38	75.54	83.96
Lomefloxacine	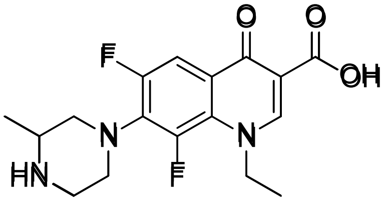	0.00	0.00	18.77
Lornoxicam	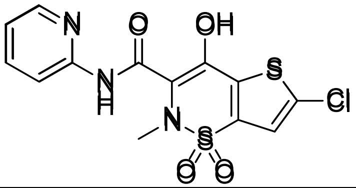	100.00	99.96	99.50
Mefloquine	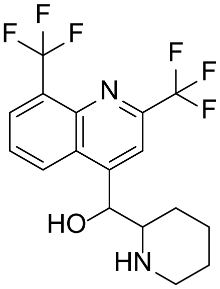	0.00	1.57	0.00
Meloxicam	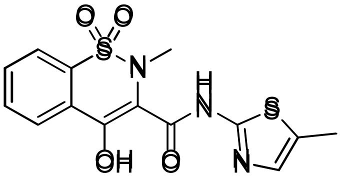	100.00	98.08	97.41

Gallopamil	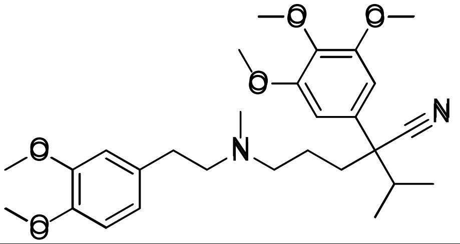	100.00	85.93	81.55
Moxifloxacin	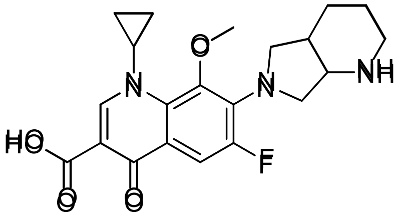	28.39	0.00	21.18
Nimesulide	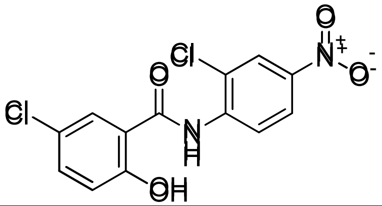	0.00	0	0.00
Norfloxacin	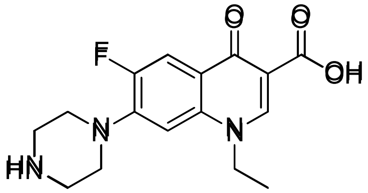	38.17	21.37	44.81
Ofloxacin	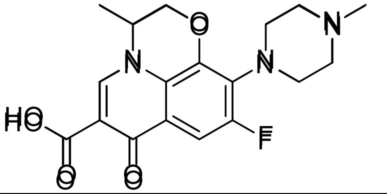	79.60	96.04	99.16
Omeprazole	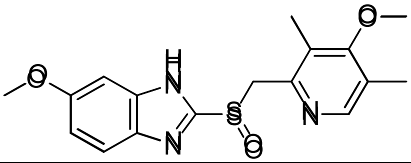	100.00	100	100.00
Oxybrupocaine	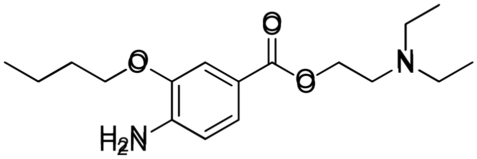	100.00	100	89.70
Oxyphenbutazone	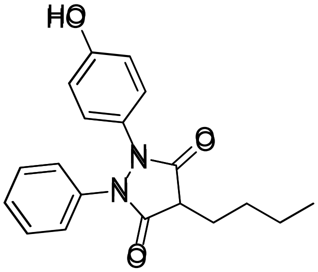	100.00	99.54	86.79

Papaverine	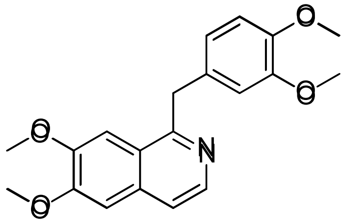	0.00	4.96	0.00
Penamidine	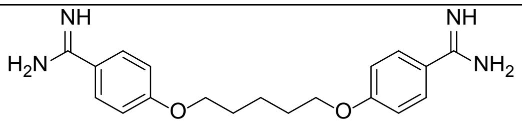	0.00	0.00	0.00
Phenserine	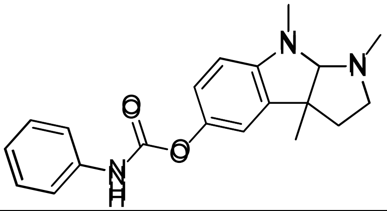	100.00	100.00	92.21
Phenylbutazone	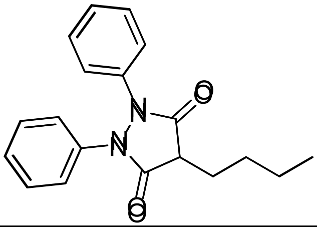	90.13	99.65	88.07
Pirenzepine	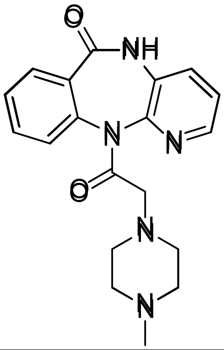	0.00	5.95	0.00
Prendisoline		0.00	0.00	10.07
Prochlorperazine	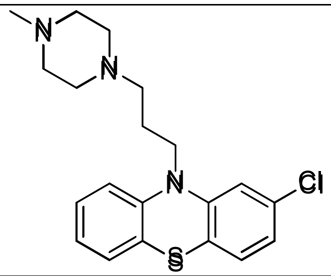	91.02	96.77	78.78
Progesterone	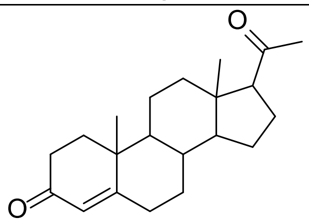	0.00	14.47	0.00

Propionylpromazine	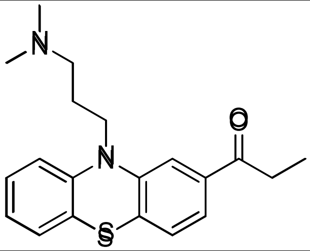	100.00	99.92	99.49
Quinacrine	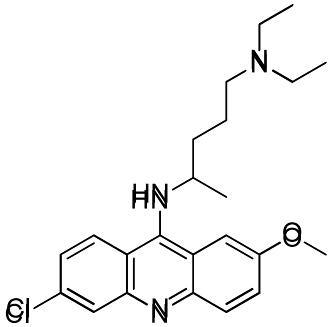	100.00	98.32	99.80
Quinine	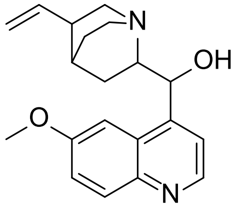	0.00	5.78	0.00
Ranitidine	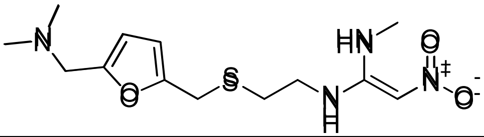	60.58	44.53	47.09
Rosuvastatin	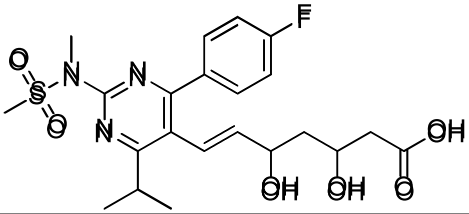	0.00	0.20	0.00
Silibinin	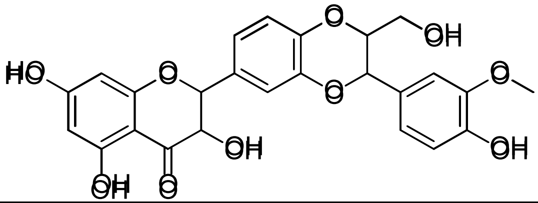	88.19	94.67	91.01
Sulfasalazine	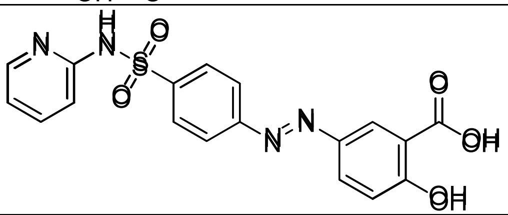	65.03	57.39	48.00

Sulindac	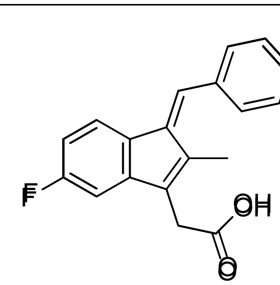	71.99	76.03	62.56
Tamoxifen	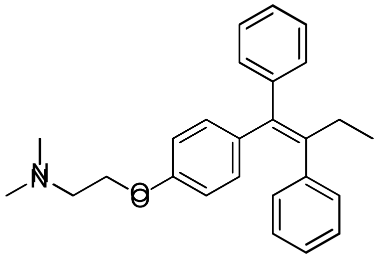	91.05	84.84	90.95
Telmisartan	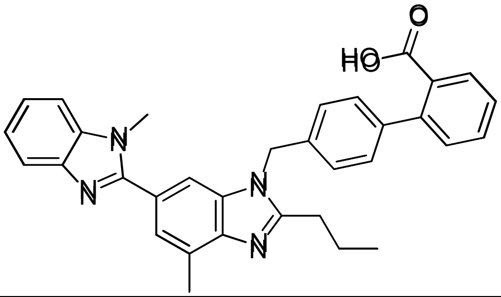	0.00	0.00	0.00
Thioridazine	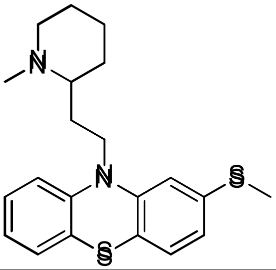	100.00	100.00	99.92
Tolnaftate	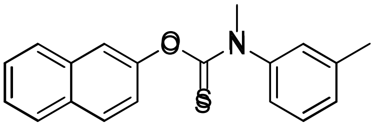	12.80	4.13	26.03
Trifluoperazine	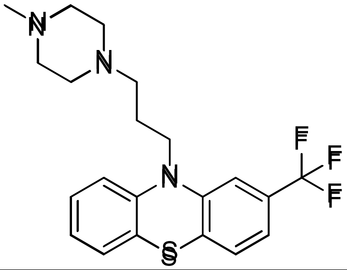	100.00	99.88	99.70

Verapamil	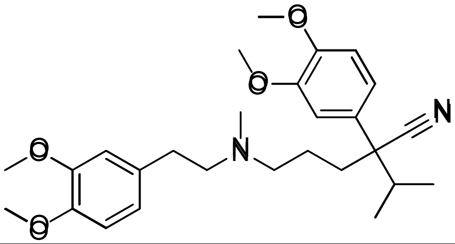	100.00	99.89	99.98
Warfarin	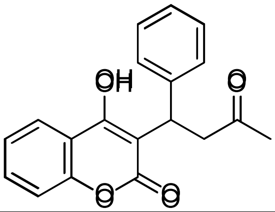	100.00	99.79	96.14
